# The taxonomic distribution of histamine-secreting bacteria in the human gut microbiome

**DOI:** 10.1186/s12864-021-08004-3

**Published:** 2021-09-26

**Authors:** Zhongyu Mou, Yiyan Yang, A. Brantley Hall, Xiaofang Jiang

**Affiliations:** 1 National Center for Biotechnology Information, National Library of Medicine, National Institutes of Health, Bethesda, MD USA; 2 Center for Bioinformatics and Computational Biology, University of Maryland, College Park, MD USA; 3 Department of Cell Biology and Molecular Genetics, University of Maryland, College Park, MD USA

**Keywords:** Histidine decarboxylase, Histamine-secreting bacteria, Human gut microbiota, Inflammatory bowel disease

## Abstract

**Background:**

Biogenic histamine plays an important role in immune response, neurotransmission, and allergic response. Although endogenous histamine production has been extensively studied, the contributions of histamine produced by the human gut microbiota have not been explored due to the absence of a systematic annotation of histamine-secreting bacteria.

**Results:**

To identify the histamine-secreting bacteria from in the human gut microbiome, we conducted a systematic search for putative histamine-secreting bacteria in 36,554 genomes from the Genome Taxonomy Database and Unified Human Gastrointestinal Genome catalog. Using bioinformatic approaches, we identified 117 putative histamine-secreting bacteria species. A new three-component decarboxylation system including two colocalized decarboxylases and one transporter was observed in histamine-secreting bacteria among three different phyla. We found significant enrichment of histamine-secreting bacteria in patients with inflammatory bowel disease but not in patients with colorectal cancer suggesting a possible association between histamine-secreting bacteria and inflammatory bowel disease.

**Conclusions:**

The findings of this study expand our knowledge of the taxonomic distribution of putative histamine-secreting bacteria in the human gut.

**Supplementary Information:**

The online version contains supplementary material available at 10.1186/s12864-021-08004-3.

## Background

Histamine is a health-relevant biogenic amine that plays important physiological roles in vascular permeability, mucus secretion, and neurotransmission via immunomodulation [1–3]. The enzyme histidine decarboxylase produces histamine via the decarboxylation of the amino acid histidine [4]. Two major families of histidine decarboxylase have been identified: pyridoxal-5′-phosphate (PLP) dependent histidine decarboxylases, which require PLP as a cofactor; and pyruvoyl-dependent histidine decarboxylases, which require a covalently bonded pyruvoyl moiety instead of PLP [5]. The decarboxylation of histidine occurs in the bacterial cytoplasm, therefore the histidine/histamine antiporter, which transports histidine into the cell and exports histamine out of the cell, is necessary for histidine decarboxylation to occur [6, 7].

While endogenously-produced histamine has been extensively studied, studies on exogenously-produced histamine have focused mostly on food-borne poisoning via histamine present in fish and dairy products [8, 9]. For example, food contaminated with a high concentration of histamine can cause neurological, gastrointestinal, and respiratory disorders [10, 11]. Histamine accumulated in food products is primarily derived from histamine-secreting bacteria, which generate histamine and other amines to maintain neutral cytoplasmic pH, allowing them to survive in acidic conditions [12]. For example, *Lactobacillus vaginalis* was found to produce histamine to maintain appropriate cytosolic pH in acidic conditions [13]. Pathogenic enteric bacteria also utilize amino acid decarboxylases to survive passage through the highly acidic gastric environment before reaching the gut. For example, the decarboxylation of L-arginine to agmatine offers *Escherichia coli* a robust acid-resistance mechanism [14, 15]. Alterations in microbiome taxonomic composition have been linked to many inflammatory diseases such as Inflammatory Bowel Disease (IBD) and asthma [16, 17]. For example, a recent study showed that histamine-secreting bacteria were increased in the gut of asthma patients [18]. Studies also demonstrated that the histamine secreted by the microbes influences immune responses within the gut via the host histamine receptor 2 and could exhibit anti-inflammatory effects [19–21].

However, our understanding of histamine-secreting bacteria and exogenous histamine production in the human gut is incomplete and limited to only a few cultured species [8, 22]. Despite the sequencing of numerous bacterial genomes, we lack a clear understanding of which gut bacteria are capable of producing and secreting histamine [23]. Therefore, a systematic functional annotation of histamine-secreting bacteria is needed to provide a comprehensive understanding of the abundance and prevalence of histamine-secreting bacteria in the human gut and to assess the role of bacterially-derived histamine in human health and disease. In this study, we conducted a systematic *in-silico* search of putative histamine-secreting bacteria in the 31,910 genomes from the Genome Taxonomy Database (GTDB) [24, 25] and the 4644 genomes in the Unified Human Gastrointestinal Genome database (UHGG) [26]. We further analyzed the relative abundance of putative histamine-secreting bacteria in metagenomic sequencing data from colorectal cancer (CRC) and inflammatory bowel disease (IBD) cohorts. Here, we aim to increase our understanding of the prevalence and abundance of histamine-secreting bacteria in the human gut microbiome and probe whether the abundance of histamine-secreting bacteria is altered in inflammatory disease.

## Results

### Histamine-secreting bacteria are sporadically distributed across six bacterial phyla

We manually collected and curated profile hidden markov models (HMM) for gene families involved in the histidine decarboxylation pathway and performed a systematic search on the proteomes from 31,910 genomes in GTDB. We identified 97 putative histamine-secreting bacteria with at least one histidine decarboxylase (*hdcA*) and one amino acid transporter (*hdcP*) localized in a gene cluster. Of these, 57 species contained the pyruvoyl-dependent histidine decarboxylase, 39 species contained the PLP-dependent histidine decarboxylase, and one species, *Plesiomonas shigelloides,* contained both pathways (Table S1). *Plesiomonas shigelloides* is a known histamine producer that causes acute diarrhea in humans after seafood consumption [27].

**Fig. 1:**
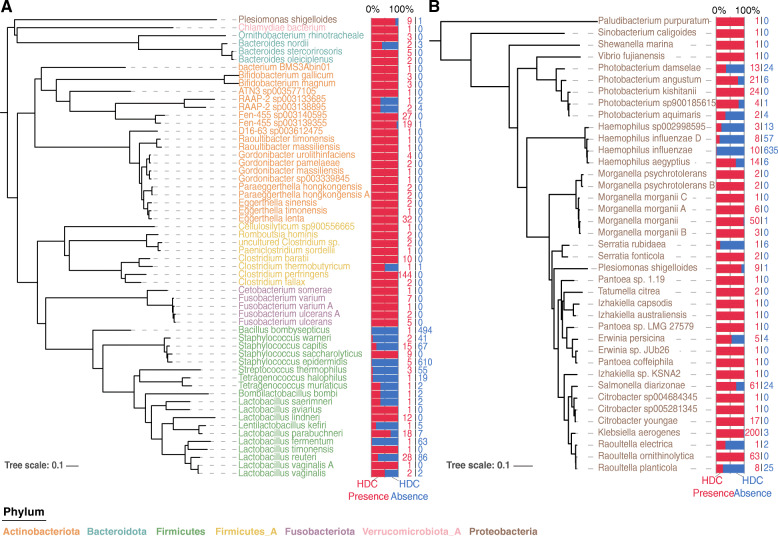
Distribution of putative histamine-secreting bacteria across the Genome Taxonomy Database (GTDB) collection. Phylogenetic trees of (A) pyruvoyl-dependent and (B) PLP-dependent putative histamine-secreting bacteria across the 31,910 genomes of the representative GTDB collection were shown. The tip label colors represent different phyla. Stacked bar charts aligned to tree tips represent the percentages of genomes with and without HDC clusters in the species. The following red and blue numbers indicate the number of genomes with HDC present and absent, respectively

The putative histamine-secreting bacteria we identified were distributed across 6 phyla. Among the identified putative histamine-secreting bacteria, the species containing the pyruvoyl-dependent histidine decarboxylase were found in 6 phyla: *Proteobacteria, Actinobacteriota, Bacteroidota, Firmicutes, Fusobacteriota, Verrucomicrobiota* (Fig. 1A). Species containing the PLP-dependent histidine decarboxylase were found only in the phylum *Proteobacteria* (Fig. 1B). *Proteobacteria* expansion is considered to be a potential indicator of gut dysbiosis, a characteristic of proinflammatory diseases such as IBD [28]. Thirty-seven of the putative histamine-secreting bacteria we computationally identified in GTDB were experimentally confirmed to be histamine producers in prior studies (Table S2), validating the search strategy used in this study. Compared to previously identified species, we have identified additional putative histamine-secreting species in the Phyla *Fusobacteriota* and *Verrucomicrobiota* including *Cetobacterium somerae*, *Fusobacterium ulcerans*, *Fusobacterium ulcerans A*, *Fusobacterium varium, Fusobacterium varium A,* and *21–14–0-10-35-9 sp002773835 (in Verrucomicrobiota).* To the best of our knowledge, no species in these two phyla has been experimentally verified to produce histamine.

Among the putative histamine-secreting bacteria in GTDB, five genera including *Bacteroides, Clostridium, Bifidobacterium, Fusobacterium, and Lactobacillus* are common in the human gut microbiota [29–32]. Therefore, we searched for histamine-secreting bacteria in the UHGG database following the same procedure as in the GTDB database. Among the 4644 species in UHGG, we identified 44 putative histamine-secreting bacteria. Of these, 35 species encode a pyruvoyl-dependent decarboxylase, 8 species encode a PLP-dependent decarboxylase, and *Plesiomonas shigelloides* encodes both pyruvoyl-dependent and PLP-dependent decarboxylases (Fig. S1, Table S3). All phyla, 21 out of 24 genera, and 33 out of 44 species of putative histamine-secreting bacteria found in UHGG were also identified in GTDB. Among the 11 species exclusively found in UHGG, five are unassigned new species and unique in UHGG, while the other 6 species are likely due to the strain-level difference between the species collected in the GTDB and UHGG database.

We further examined the validity of the decarboxylases with structural modeling. We performed 3D protein homology modeling of pyruvoyl-dependent *hdcA* based on a high-resolution crystal structure (*Lactobacillus saerimneri* PDB: 1PYA) [33]. The backbone RMSD scores of pyruvoyl-dependent *hdcA* models were within 1 Å when comparing with the crystalline template (Fig. S2), indicating significant structural similarity between the putative histamine-secreting bacteria and the known histamine-secreting bacterium *Lactobacillus saerimneri*. Figure S2A shows the similarity between the model of *hdcA* of *Clostridium perfringens* and the template *Lactobacillus saerimneri 30a* (PDB: 1PYA) with a backbone RMSD of only 0.61 Å. The experimentally confirmed key residues in the active site of *hdcA* in *Lactobacillus saerimneri 30a* were illustrated in Fig. S2B including the Ser-81 and Ser-82 as the autocleavage pair [5], Ile-59 as the “lid” on the substrate-binding pocket [6], the Asp-198 and Asp-53 pair as the pH-regulating bridge [7], and the Tyr-62 and Asp-63 as the ligand-binding residues [33, 34]. The high structural similarity and identical key residues, as shown in Fig. S2C, strengthens the evidence of the genomic potential to produce histamine in these bacteria. We did not perform modeling on *hdcP* and PLP-dependent *hdcA* due to the lack of crystal structures and established enzymatic mechanisms for these proteins.

The ability to produce and secrete histamine is not a core function in all identified species. Out of the 97 species with a putative histamine-secretion operon in GTDB, 21 species belong to a clade where more than 50% of the strains lack the operon. These strain-specific histamine-secreting bacteria were predominantly observed in the phyla *Firmicutes* and *Proteobacteria*. Within the Proteobacteria, the genomic potential for histamine-secretion was found to be strain-specific in the families *Pasteurellaceae*, *Vibrionaceae*, and *Enterobacteriaceae*. In the Firmicutes, the genomic potential of histamine secretion was also found highly strain-specific in the *Bacillaceae, Enterococcaceae, Lactobacillaceae, Staphylococcaceae,* and *Streptococcaceae* families (Table S1). Species such as *Streptococcus thermophilus*, *Staphylococcus warneri*, *Lactobacillus parabuchneri*, and *Lactobacillus reuteri* have been previously reported as strain-specific in terms of histamine production [35–38], which agrees with the results of our computational search.

**Fig. 2:**
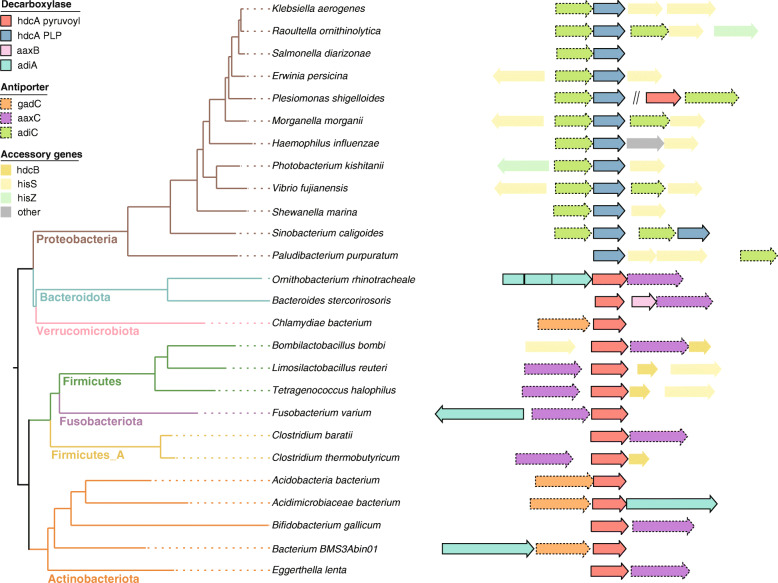
Representative histidine decarboxylase gene clusters for putative histamine-secreting bacteria. Two types of bacterial histidine decarboxylases were labeled as *hdcA* pyruvoyl and *hdcA* PLP respectively. Two arginine decarboxylases were identified in some histidine decarboxylase species including aaxB and adiA. Three different types of amino acid antiporters in histidine decarboxylase clusters (hdcP) were labelled as gadC, aaxC and adiC. The phylogenetic tree was a subtree extracted from a pre-built GTDB phylogenomic tree based on 120 bacterial marker genes. Gene clusters along with the tree were visualized by ggtree (version 2.5.1) [43]. The break symbol (double slash mark) was placed between two distant histidine decarboxylase gene clusters for species *Plesiomonas shigelloides*

The strain-specificity of histamine-secretion may be due to the frequent loss of the histamine-secretion genes during evolution. Strain-specific gene loss is a common reason for the functional variation between different strains of the same species [39–42]. The strain-specificity could also be attributed to the horizontal gene transfer of genes encoding histamine-secretion pathways by mobile genetic elements. For example, while the histidine decarboxylase gene cluster is located on the chromosome of the *Lactobacillus reuteri* JCM 1112 strain, it is located on the pLRI01 plasmid of the *Lactobacillus reuteri* I5007 strain. The histidine decarboxylase gene cluster on the plasmid can be mobilized via conjugation and might have been horizontally transferred to *Clostridium thermobutyricum* (GCF_000371465.1). In *Clostridium thermobutyricum,* the histidine decarboxylase cluster is both phylogenetically closer (Fig. S3) and syntenically more similar (Fig. 2) to the histidine decarboxylase cluster in the plasmid of *Lactobacillaceae* than that in other *Clostridiaceae*. Such mobility of histidine decarboxylase gene clusters was also reported in a study on the species of *Lactobacillus parabuchneri* [44], supporting the hypothesis that mobile genetic elements could play an important role in the strain-specificity of histamine-secreting traits.

### New gene clusters observed in the putative histamine-secreting bacteria

Three different types of antiporters (*hdcP*) were found in the histidine decarboxylase gene clusters. These antiporters were annotated as the glutamate/gamma-aminobutyrate antiporter “*gadC*”, and the arginine/agmatine antiporters “*aaxC*” and “*adiC*”. The *gadC* and *aaxC* genes were observed with the pyruvoyl-dependent *hdcA* whereas adiC was found with the PLP-dependent *hdcA*. The only exception was seen in *Plesiomonas shigelloides*, which has both the pyruvoyl-dependent and PLP-dependent pathways, where the *adiC* antiporter was found in the gene cluster of both the PLP-dependent and pyruvoyl-dependent *hdcA*. At the phylum level, in *Bacteroidota, Firmicutes, and Fusobacteriota*, histidine decarboxylase was adjacent to *aaxC*; in *Verrucomicrobiota,* histidine decarboxylase was adjacent to *gadC*; and in *Actinobacteroiodota,* histidine decarboxylase was adjacent to either *aaxC* or gadC (Fig. S3).

Duplications of the antiporters were observed in some *Proteobacteria* where antiporter genes were found both upstream and downstream of the PLP-dependent *hdcA*. In addition to *hdcA* and *hdcP*, *hdcB*, which catalyzes the maturation of pyruvoyl-dependent *hdcA*, was occasionally found in close proximity to *hdcA* as seen in *Streptococcus thermophilus* CHCC1524 [45]. The genes, *hisRS*, which encode histidyl-tRNA synthetase were also found in the histidine decarboxylase cluster [46–48]. Throughout our search, the *hdcA*/antiporter/*hdcB* cluster was observed in *Firmicutes* such as *Clostridium thermobutyricum* and *Streptococcus thermophilus*. The *hdcA*/*hdcB*/antiporter/*hisRS* cluster was also observed in *Firmicutes* such as *Tetragenococcus halophilus* and *Lactobacillus reuteri.* The *hdcA*/antiporter/*hisRS* cluster was observed in *Proteobacteria* such as *Klebsiella aerogenes* and *Shewanella marina.*

In several phyla of the pyruvoyl-dependent histamine-secreting bacteria, we found a two-decarboxylase, one-transporter three-component decarboxylation system (Fig. 2) composed of arginine decarboxylase, histidine decarboxylase, and an antiporter. This arginine/histidine system was similar to the lysine/ornithine three-component decarboxylation system in *Lactobacillus saerimneri 30a* [49], the first reported bacterial three-component decarboxylation system. A difference between the arginine/histidine system found in histamine-secreting bacteria and the lysine/ornithine system found in *Lactobacillus saerimneri 30a* is that gene components of the histidine/arginine system were adjacent to each other, while the genes for the lysine/ornithine system were 24 kb apart.

The two decarboxylases in the three-component histidine/arginine system can utilize different enzymatic mechanisms and cofactors. Similar to histidine decarboxylase, two types of arginine decarboxylases have been previously identified, a PLP-dependent arginine decarboxylase (*adiAYC*) and a pyruvoyl-dependent arginine decarboxylase (*aaxABC*) [50]. In the histidine/arginine systems, the histidine decarboxylases were pyruvoyl-dependent while the arginine decarboxylases were either pyruvoyl-dependent (*aaxB*) or PLP-dependent (*adiA*). For example, in *Actinobacteriota* and *Fusobacteriota*, the adiA (PLP-dependent)/antiporter/*hdcA* (pyruvoyl-dependent) cluster was observed, while in *Bacteroidota*, both the adiA/antiporter/*hdcA* (*Ornithobacterium rhinotracheale*) and *aaxB*/antiporter/*hdcA* (*Bacteroides stercorirosoris*) clusters were observed.

### Putative histamine-secreting bacteria were significantly enriched in inflammatory bowel disease patients but not in colorectal cancer patients compared to healthy controls

**Fig. 3:**
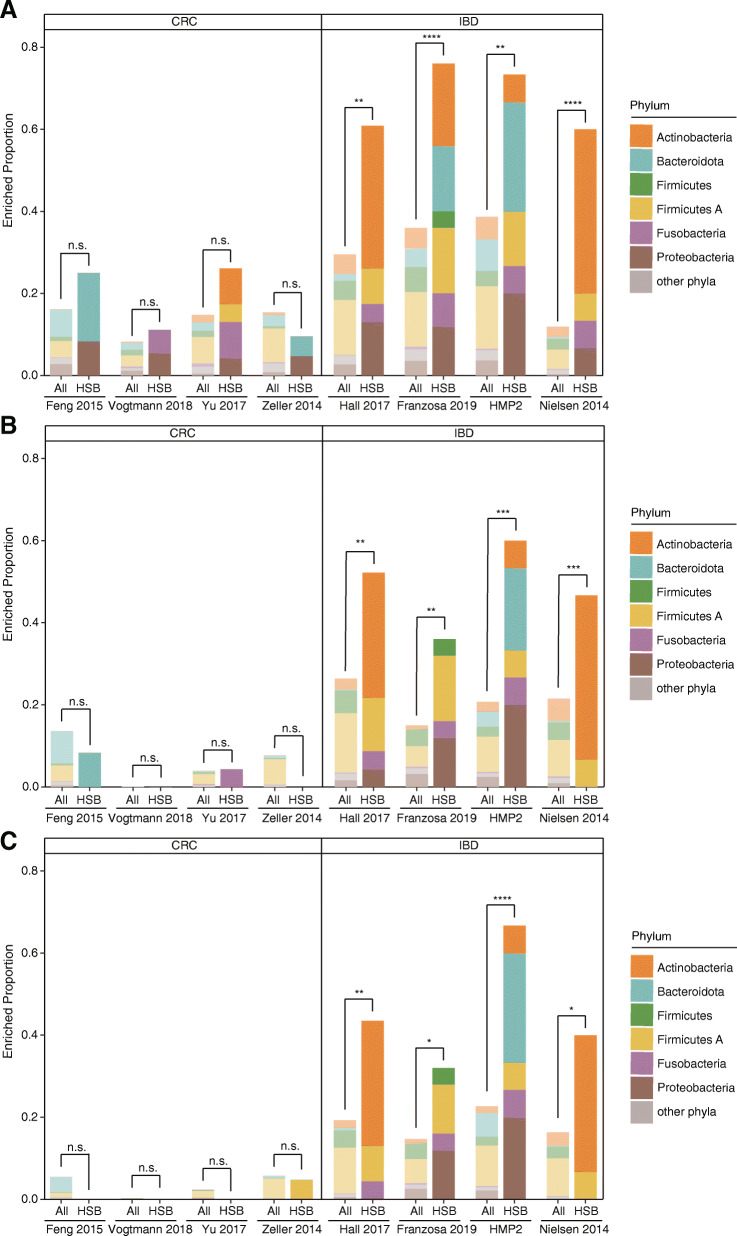
Differential abundance analysis of putative histamine-secreting bacteria. The analysis was performed on stool metagenomic samples between patients with colorectal cancer (CRC), inflammatory bowel disease (IBD) and healthy controls by DESeq2 (A), MaAslin2 (B) and LefSe (C). Relative abundances (counts) were calculated using Kraken2 (2.0.8-beta) (see methods section for more details). The criteria for enriched species for DEseq2 are log2FC > 1 (the Integrative Human Microbiome Project, i.e. HMP2 > 0.5) and q < 0.05, for MaAsLin2 are coef > 0.2 (HMP2 > 0) and q < 0.05, and for LefSe are LDA > 2 and q < 0.001. The statistical differences between all bacteria and histamine-secreting bacteria (HSB) were determined by one-tailed two-proportion Z-test with continuity correction and the *p* values were converted to asterisks (n.s. for *p* > 0.05; * for *p* ≤ 0.05; ** for *p* ≤ 0.01; *** for *p* ≤ 0.001 and **** for *p* ≤ 0.0001). Number of samples by disease state for each study are: Feng 2015 [51]: control = 61, CRC = 46; Vogtmann 2016 [52]: control = 52, CRC = 52; Yu 2017 [53]: control = 75, CRC = 53; Zeller 2014 [54]: control = 66, CRC = 91; Hall 2017 [55]: control = 74, IBD = 188; Franzosa 2019 (discovery cohorts) [56]: control = 34, IBD = 121; HMP2 [57, 58]: control = 429, IBD = 1209; Nielsen 0214 [59]: control = 248, IBD = 148. See also Table S4 for the enrichment of individual species and Table S5 for the detailed results of Z-test

Analyzing 2451 stool metagenomes from IBD patients and 506 stool metagenomes from CRC patients, we found the putative histamine-secreting bacteria identified in UHGG were significantly enriched in IBD (Ulcerative colitis and Crohn’s disease combined) patients but not in CRC patients. We identified UHGG species with differential abundance between the patients and healthy controls in eight studies, including four IBD studies and four CRC studies. To avoid bias caused by detection methods, we used three methods, namely DESeq2, MaAsLin2, and LEfSe to perform the differential abundance analysis. We then performed one-tailed two-proportion Z-tests with continuity correction to test the hypothesis that histamine secreting species have a higher probability to be positively associated with disease. In the IBD studies, we found that putative histamine-secreting bacteria were significantly enriched when compared to all gut species through Z-tests with *p* < 0.05 in all four studies and by all three statistical methods (Fig. 3, Table S4-S5). The results from DESeq2, MaAsLin2, and LEfSe generally agreed well with each other, with DESeq2 being the most relaxed method in determining enrichment. Unlike IBD studies, no significant differential abundance was observed in putative histamine-secreting bacteria when compared to the overall gut species in any CRC studies by any method.

The enrichment of histamine-secreting species was not attributed to a single taxon and we found the enriched species in IBD studies to be highly cohort-specific. For example, in the cohort in Hall et al. 2017, histamine-secreting bacteria were mainly enriched in *Actinobacteriota* and slightly enriched in *Firmicutes.* For Franzosa et al. 2019, histamine-secreting bacteria were mainly enriched in *Firmicutes* and *Proteobacteria* (Fig. 3, Table S4)*.* For HMP2, histamine-secreting bacteria were mainly enriched in *Bacteroidiota* and *Proteobacteria* and for Nielsen et al. 2014, histamine-secreting bacteria were enriched in *Actinobacteriota* (Fig. 3, Table S4). In addition, we analyzed the abundance of histidine decarboxylase operon in IBD and CRC studies. Consistent with the results of differential abundance analysis of putative histamine-secreting bacteria, a similar trend was found that HDC gene clusters were enriched in IBD studies. Our result indicated the operon abundance was significantly enriched in all of the IBD studies including Franzosa et al. 2019, Hall et al. 2017, HMP2 and Nielson et al. 2014, and one of the CRC studies, namely Yu et al. 2017 (Fig. S4).

## Discussion

In this study, we performed a systematic search for histamine secreting operons in species from the human gut microbiota. In total, we identified 117 species with the genomic potential to secrete histamine. We identified a few discrepancies in the histamine-secreting bacteria between our systematic search and previous experimental studies (Table S2). Several reasons could contribute to the discrepancies. First, the putative histamine-secreting operon was highly strain-specific in a number of histamine-secreting species [36]. The difference could be due to different strains used in this study and previous work. Second, we focused on the well-known pyruvoyl-dependent and PLP-dependent histidine decarboxylases and this approach might miss newly discovered histidine decarboxylases [60]. What’s more, the techniques to detect histamine produced by histamine-secreting bacteria varied between studies [8, 61]. For example, in *Citrobacter freundii,* 21 out of 21 strains were classified as positive for histamine-secretion by Niven’s agar method but only 1 out of 21 were classified as positive for histamine-secretion by PCR [61]. The Niven’s agar is a differential growth medium with a pH indicator. Bacteria that grow and increase the pH of the Niven’s agar are inferred to be as histamine-secreting bacteria. This method has a high false-positive rate largely due to the fact that bacteria can secrete alkaline other than histamine. The PCR based method is subject to primer bias which can lead to false negative results. The possible inaccuracy in the histamine detection methods has likely led to non-histamine-secreting bacteria mislabeled as histamine-secreting and vice versa. Further studies may include experimental evaluation of selected representatives of the putative histamine-secreting bacteria identified in this study, especially at some health-related taxonomic levels where no histamine-secreting bacteria have been described and explored before.

In this study, we identified that putative histamine-secreting bacteria were significantly enriched in IBD patients. Combined with the previous knowledge that histamine-secreting bacteria were found to be increased in the gut communities of asthma patients [18], it suggests a possible association between histamine-secreting bacteria and inflammatory diseases. Because the lumenal environment is more acidic in the small intestine than in the colon, the genomic potential of histamine-secretion may be expressed in the small intestine but not in the colon. As a result, the presence of putative histamine-secreting bacteria in prominent families found in the small intestines, such as *Enterobacteriaceae* and *Lactobacillaceae*, may have significant immunological implications (Table S3) [62]. The deeper relationships between histamine-secreting bacteria and host in terms of inflammatory and immunological diseases are yet to be elucidated.

To the best of our knowledge, the histidine/arginine decarboxylation system observed in a number of histamine-secreting bacteria from three phyla is the first three-component decarboxylation system identified in a cluster of colocalized genes. Our findings suggest that some amino acid antiporters are likely capable of transporting multiple amino acids with similar structural and chemical properties. The capability to transport multiple amino acids using a single antiporter may increase the robustness and efficiency of acid resistance in the bacteria. Further studies may include the exploration of other three-component or multi-component decarboxylation systems other than the histidine/arginine and lysine/ornithine systems.

## Conclusions

In conclusion, we have systematically annotated bacterial histidine decarboxylase in two large databases. 117 putative histamine-secreting bacteria species were identified throughout GTDB and UHGG including those in *Fusobacteriota* and *Verrucomicrobiota* where no histamine-secreting bacteria had been previously described*.* We have identified a novel three-component decarboxylase system which contains an arginine decarboxylase, a histidine decarboxylase and an antiporter. Differential abundance analysis showed that the putative histamine-secreting bacteria were significantly enriched in IBD patients but not in CRC patients. Our findings would expand the knowledge and provide a comprehensive understanding of histamine-secreting bacteria in the human gut and facilitate advances in potential therapeutic targets toward histamine-related inflammatory and immunological diseases.

## Methods

### Histidine decarboxylase operon identification

The Unified Human Gastrointestinal Genome (UHGG) [26] and Genome Taxonomy Database (GTDB) (release 95) were used as the databases for histamine-secreting bacteria identification [24, 25]. To identify PLP-dependent histidine decarboxylases, we performed a multiple sequence alignment of known PLP-dependent histidine decarboxylase genes (WP_191935110.1,WP_068969528.1,WP_152135723.1 and WP_136342781.1) with MUSCLE 3.8.31 [63] and then built a customized profile HMM with hmmbuild from HMMER 3.3.1 package [64]. This customized profile was used to search query protein databases using hmmscan. Hits with e-value 1e-100 or less were selected and manually curated and annotated as PLP-dependent histidine decarboxylases. To identify pyruvoyl-dependent histidine decarboxylases, we searched the query database using hmmscan against Pfam profile PF02329 (Histidine carboxylase PI chain) with cutoff e-value 1e-40. We searched for amino acid antiporters using hmmscan against PANTHER profile PTHR42770 with cutoff e-value 1e-40. The genomic loci where histidine decarboxylase and antiporters are present in the same gene neighborhood (less than three genes away) were identified as histamine secretion gene clusters. The gene structure in putative histamine-secreting bacteria was then manually screened in Geneious Prime 2021.0.3 [65].

### Histidine decarboxylase 3D modeling

For the putative pyruvoyl-dependent decarboxylase *hdcA*, the amino acid sequences of histamine-secreting bacteria from UHGG and GTDB were aligned with Clustal Omega [66, 67]. BLAST and HHpred servers were used to identify potential structural templates from the Protein Data Bank [68, 69]. We used the 2.5 Å X-ray crystal structure of a bacterial HhdA from *Lactobacillus saerimneri 30a* (UniProt ID P00862, PDB entry 1PYA) as the sole template [33]. Due to the inter-oligomeric contacts and active site, a trimer C3 symmetry was defined based on 1PYA and used in modeling. The 3mer and 9mer fragment files were obtained from the Robetta server (http://old.robetta.org/). RosettaCM was then employed to generate at least 1000 models of each protein. We selected the top ten models based on the Rosetta energy and, among these ten, selected the lowest backbone RMSD to the 1PYA trimer crystal structure as the final protein models. Unlike pyruvoyl-dependent *hdcA* with auto-cleavage and multi-chain modeling with only one good template, no crystal structure was reported for bacterial *hdcP* and the PLP-dependent *hdcA* therefore no model was built upon *hdcP* and the PLP-dependent *hdcA*.

### Phylogenetic analysis

To construct the phylogeny of pyruvoyl-dependent *hdcA* genes, a multiple sequence alignment was performed by Clustal Omega v1.2.3 with fifty-eight pyruvoyl *hdcA* proteins [66] and trimmed with trimAl v1.2 on strictplus mode [70]. A maximum-likelihood phylogenetic tree was inferred by IQ-TREE v2.1.2 using its suggested LG + I + G4 model with 1000 ultrafast bootstrap replicates [71]. This tree was rooted using the Minimal Ancestor Deviation (MAD) method via mad v2.2 [72] and was visualized and annotated using iTOL v5 (https://itol.embl.de/) [73].

### Metagenomic data processing

The raw sequencing reads of metagenomic samples used in this study were downloaded and extracted using National Center for Biotechnology Information (NCBI)‘s SRA Toolkit [74] v2.10.9 under the accession numbers of PRJEB1220 [59], HMP2 (PRJNA398089 [58], PRJNA389280 [57]), PRJNA385949 [55], and PRJNA400072 [56] for IBD studies and accession numbers for studies are PRJEB6070 [54], PRJEB7774 [51], PRJEB12449 [52], and PRJEB10878 [53] for colorectal cancer (CRC) studies. Quality control and adapter trimming of the fastq sequence files were done with Trim Galore wrapper v0.6.6 [75]. Quality trimmed sequences were screened against the human assembly NCBI build 37 (hg19) using Bowtie2 v2.4.2 alignment software [76] and Samtools v1.11 [77] was then used to remove human genome contamination unmapped sequence from SAM files. Taxonomic assignment of filtered reads was performed using Kraken2 v2.0.8-beta (with default settings) [78] against a pre-built database of the UHGG catalog (http://ftp.ebi.ac.uk/pub/databases/metagenomics/mgnify_genomes/human-gut/v1.0/uhgg_kraken2-db/) [26].

### Differential taxonomic abundance analysis

The kraken species-level abundance outputs and metadata were imported into the phyloseq R package (v.1.34.0) for analyses [79]. Samples with sequencing depth less than 1 million counts were excluded. Rare taxa with a relative abundance of less than 0.01% across 10% of all samples were filtered. To minimize the bias of a single method and report a robust analysis, we employed three commonly used statistical methods including DESeq2 [80], Multivariate Association with Linear Models 2 (MaAsLin2) [81], and Linear discriminant analysis effect size (LEfSe) [82] for differential abundance analysis. For DEseq2, the “local” method was employed to estimate dispersion, and the “poscounts” size factor estimator was employed in the normalization step to exclude the zeros when calculating the geometric mean. The DEseq2 “poscounts” normalized counts were used as input for LEfSe. For MaAsLin2, the trimmed mean of M-values (TMM) method was applied as the normalization method based on its satisfactory performance in a recent benchmark [83]. We defined species with more than 50% of the strains encoding histidine decarboxylase gene clusters as histamine-secreting species. One-tailed, two-proportion Z-tests with continuity correction were performed to compare the difference between the proportion of disease-associated species in defined histamine secreting species and the proportion of disease-associated species in all species.

### Histidine decarboxylase operon abundance estimation and analysis

We created a set of reference genomes mainly from the representative genomes from the UHGG. If the representative genome for a species in the UHGG does not contain the histidine decarboxylase operon and a different genome of the same species has, we replaced it with the genome with the histidine decarboxylase operon instead. We aligned the cleaned metagenomic reads to the reference genomes using bowtie2 [76]. Samples with total read counts less than 1 million were filtered out. The abundance of histidine decarboxylase operon was estimated by the number of per million reads mapped to the histidine decarboxylase operon divided by the total number reads mapped to the reference genomes (namely measured in counts per million reads mapped, CPM). One sided Wilcoxon’s rank-sum test with continuity correction was performed to test the difference of the histidine decarboxylase operon abundance in the patient’s sample and that in the healthy control.

## Supplementary Information

**Additional file 1 Table S1. Putative histamine-secreting bacteria (HSB) species identified in GTDB. (XLSX 53 kb)**

**Additional file 2 Table S2. Experimentally putative verified histamine-secreting bacteria (HSB) species. (XLSX 18 kb)**

**Additional file 3 Table S3. Putative histamine-secreting bacteria (HSB) species identified in UHGG. (XLSX 28 kb)**

**Additional file 4 Table S4. Enriched HSB of UHGG in IBD studies. This table corresponds to the species in** Fig. 3**of the main text. (XLSX 13 kb)**

**Additional file 5 Table S5. One-tailed two-proportion Z tests for enriched HSB of UHGG in IBD and CRC studies. This table corresponds to the enriched proportion in** Fig. 3**of the main text. (XLSX 11 kb)**

**Additional file 6 (DOCX 1547 kb)**

## Data Availability

Genomes analysed during the current study are available in the GTDB, https://data.ace.uq.edu.au/public/gtdb/data/releases/release95/95.0/ and UHGG, http://ftp.ebi.ac.uk/pub/databases/metagenomics/mgnify_genomes/human-gut/v1.0/uhgg_catalogue/. The raw sequencing reads of metagenomic samples were downloaded from Sequence Read Archive (SRA) from the National Center for Biotechnology Information (NCBI) database (SRA accession numbers of PRJEB1220, PRJNA389280, PRJNA398089, PRJNA385949, and PRJNA400072 for IBD studies and PRJEB6070, PRJEB7774, PRJEB12449, and PRJEB10878 for CRC studies). All data generated or analyzed during this study are included in this published article (and its supplementary information files).

## Abbreviations

CRC: Colorectal cancer
GTDB: Genome Taxonomy Database
HDC: Histidine decarboxylase
HMP2: Integrative Human Microbiome Project
HSB: Histamine-secreting bacteria
IBD: Inflammatory bowel disease
PDB: Protein data bank
PLP: Pyridoxal-5′-phosphate
RMSD: Root mean square deviation
UHGG: Unified Human Gastrointestinal Genome
