# Supplementary material for: The taxonomic distribution of histamine-secreting bacteria in the human gut microbiome
Source: BMC Genomics. 2021 Sep 26;22:695. doi: 10.1186/s12864-021-08004-3 (PMC8465708; doi:10.1186/s12864-021-08004-3)
Supplement: Supplementary file 6 — Additional file 6 (DOCX 1547 kb) [file 12864_2021_8004_MOESM6_ESM.docx]

**Supporting Information for**

**The taxonomic distribution of histamine-secreting bacteria in the human gut microbiome**

**Zhongyu Mou, Yiyan Yang, A. Brantley Hall, Xiaofang Jiang**

**Supplementary Tables**

**Table S1. Putative histamine-secreting bacteria (HSB) species identified in GTDB.**

see additional file: Table_S1.xlsx

**Table S2. Experimentally putative verified histamine-secreting bacteria (HSB) species.**

see additional file: Table_S2.xlsx

**Table S3. Putative histamine-secreting bacteria (HSB) species identified in UHGG.**

see additional file: Table_S3.xlsx

**Table S4. Enriched HSB of UHGG in IBD studies. This table corresponds to the species in Figure 3 of the main text.**

see additional file: Table_S4.xlsx

**Table S5. One-tailed two-proportion Z tests for enriched HSB of UHGG in IBD and CRC studies. This table corresponds to the enriched proportion in Figure 3 of the main text.**

see additional file: Table_S5.xlsx

**Supplementary Figures**


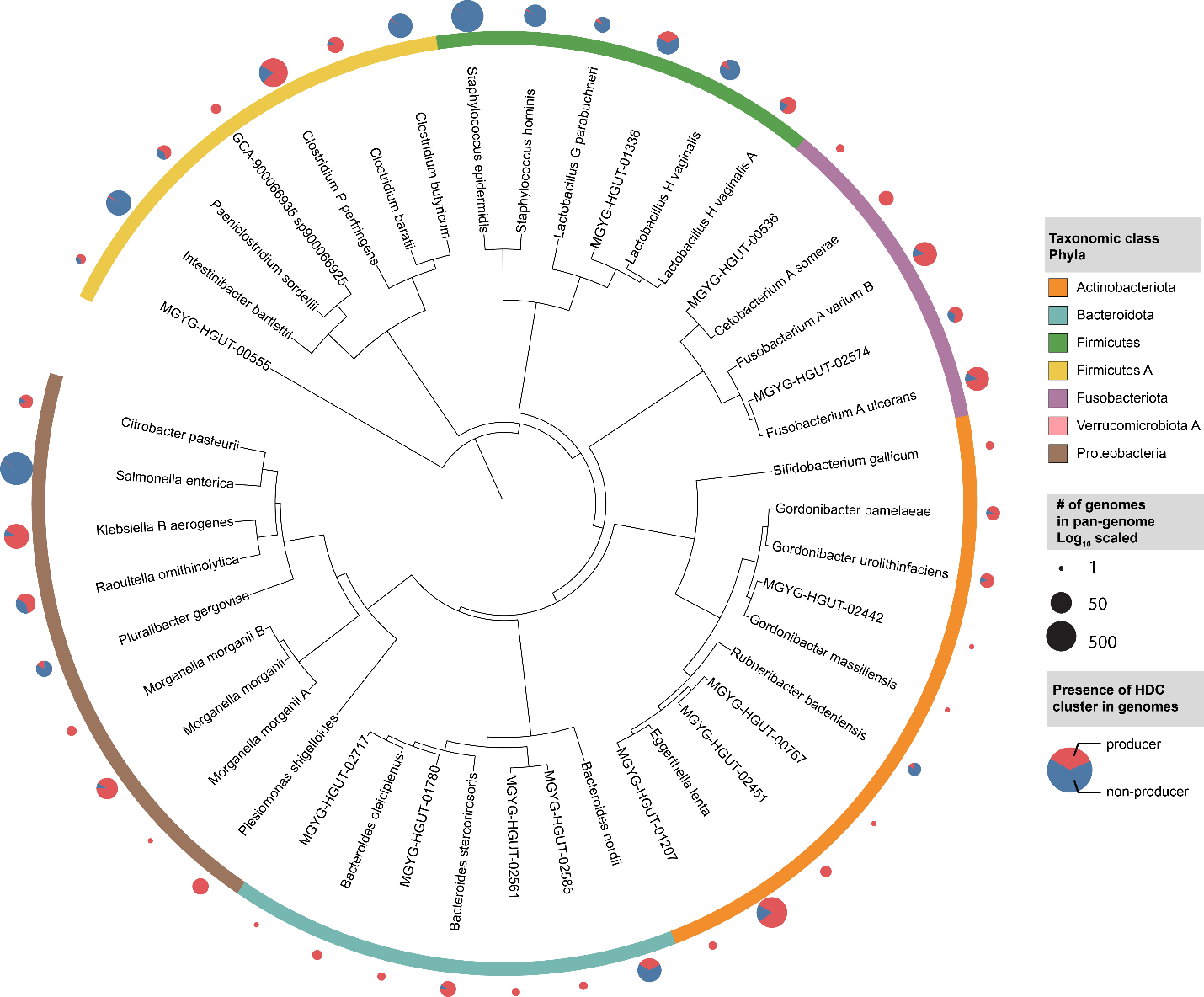
**Figure S1. Distribution of putative histamine-secreting bacteria across the Unified Human Gastrointestinal Genome (UHGG) collection.** A phylogenetic tree of pyruvoyl-dependent histamine-secreting bacteria across the 4,644 genomes of the representative UHGG collection. Branch length was turned off. Phyla are labeled by color strip. Fill colors of the pie charts correspond to the strain-specificity of histamine-secreting bacteria and the relative size of the pie charts represents the total number of strains in species pan genomes.
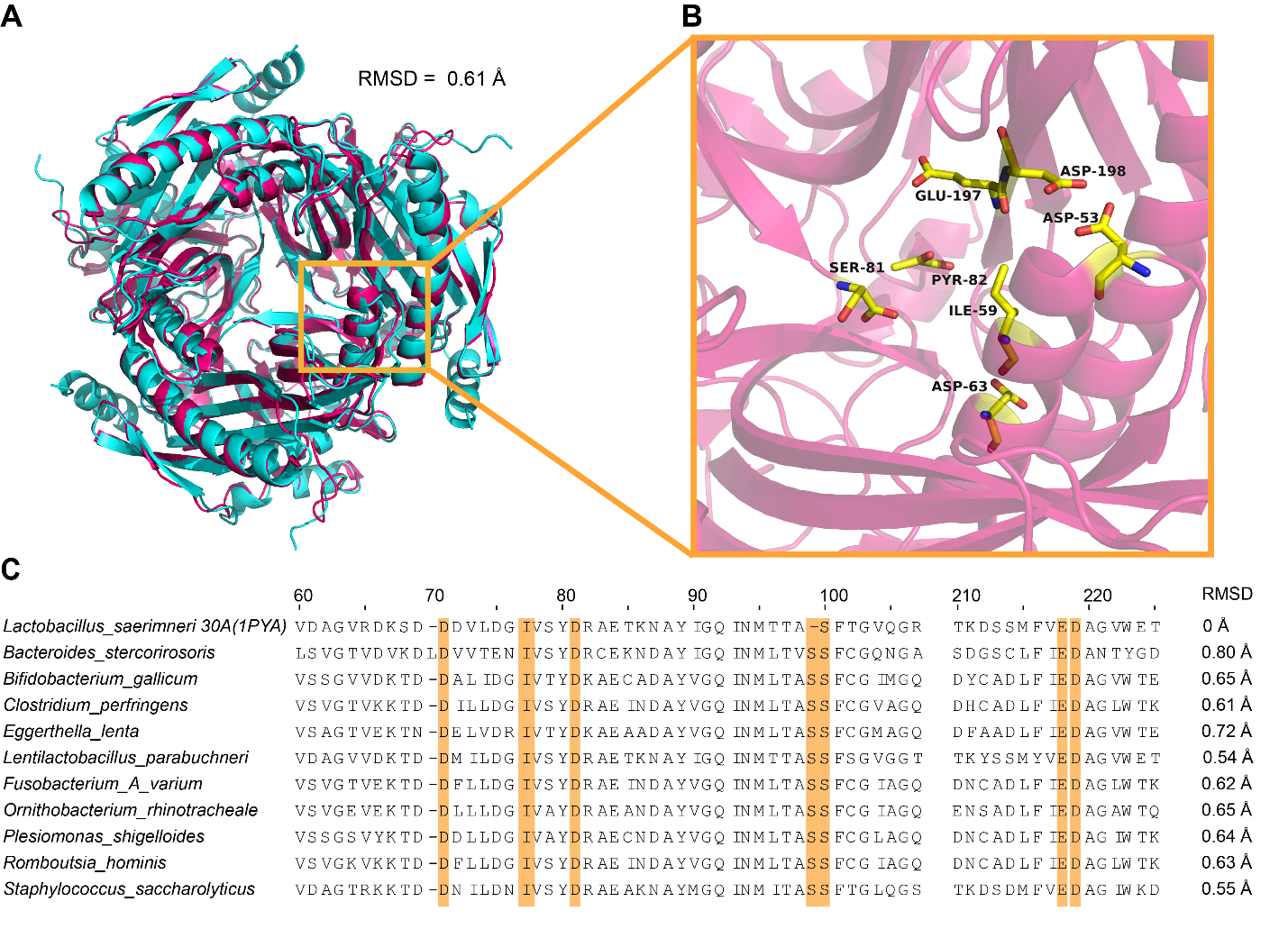


**Figure S2.** **Alignment between putative histidine decarboxylases and the crystal structure and conserved active residues.** (A) The alignment between template *Lactobacillus saerimneri 30a* (PDB: 1PYA) in pink and the homology 3D model of HSB Clostridium perfringens in cyan. Backbone root mean square deviation (RMSD) using Pymol align method is shown. (B) Key residues of *Lactobacillus saerimneri 30a.* (C) Multiple sequence alignment between selected hdcA genes in putative pyruvoyl-dependent HSB in GTDB and *Lactobacillus saerimneri 30a.* Key residues are highlighted.

**
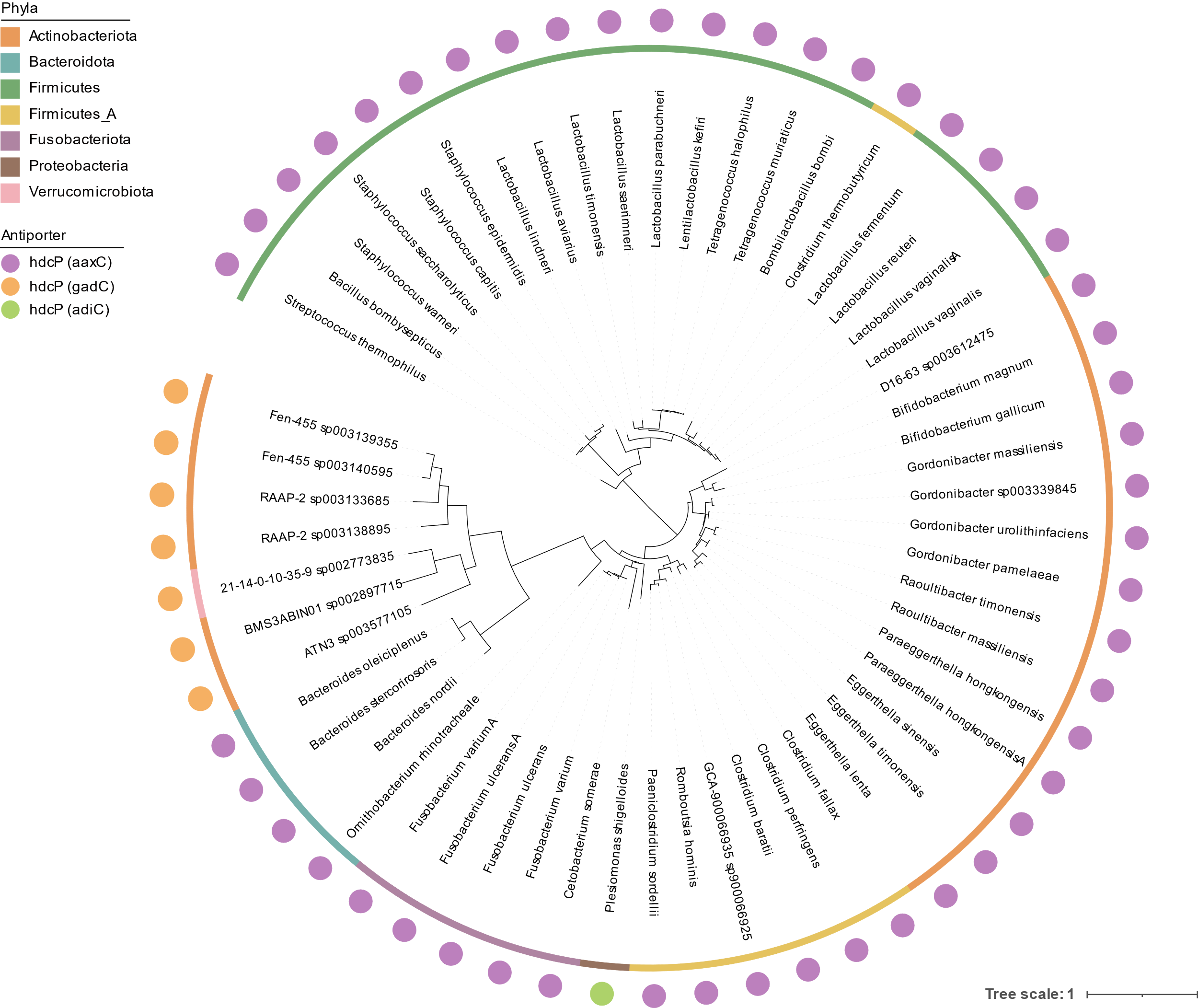
**

**Figure S3. Phylogenetic tree of pyruvyl-dependent hdcA of putative histamine-secreting bacteria (HSB) in GTDB.** Phyla are labeled by color strip. The corresponding antiporters were labeled by color circles.


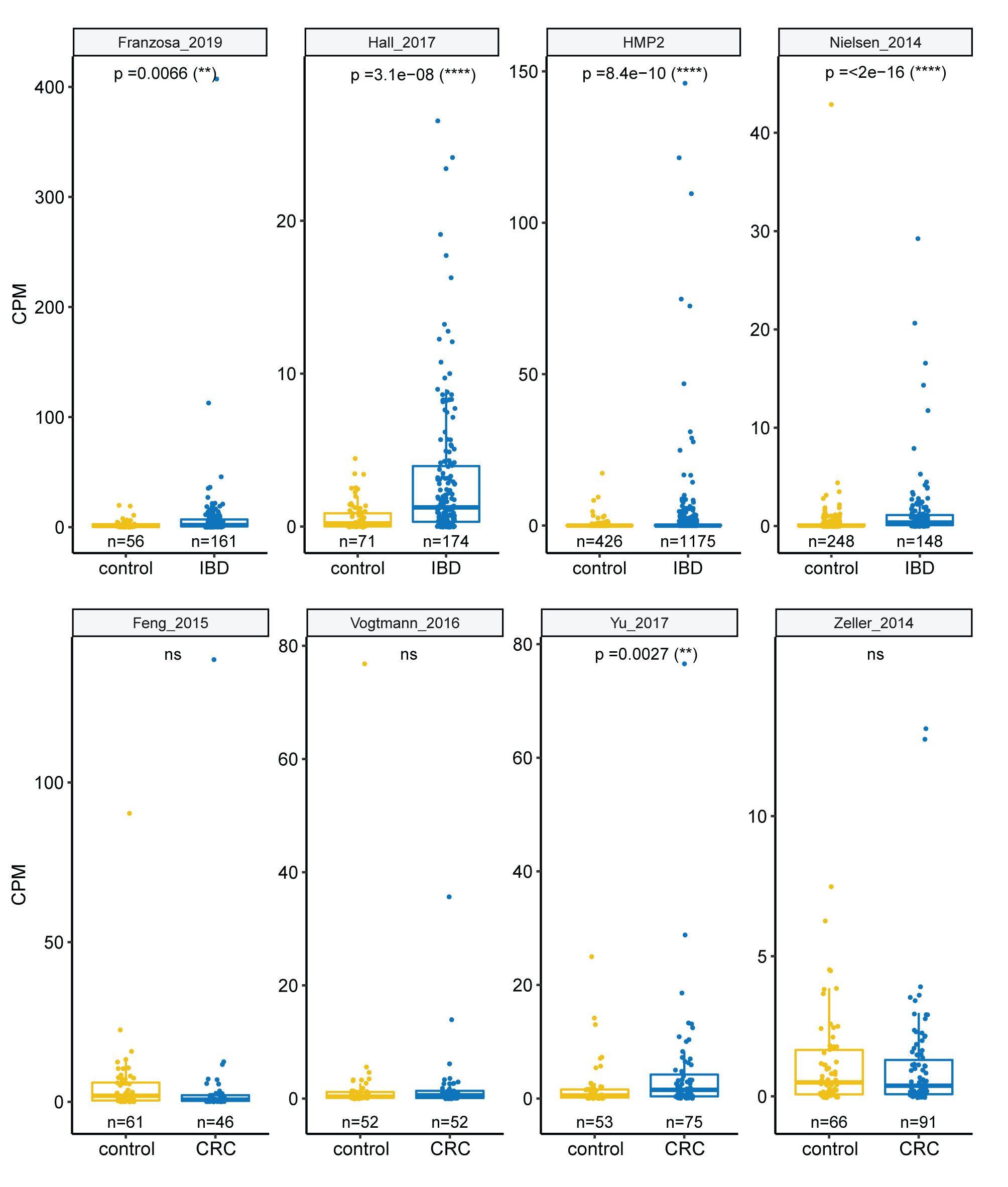


**Figure S4. Histidine decarboxylase operon abundance for IBD (top panel) and CRC (bottom panel) studies.** HDC operon abundance was measured in units of counts per million reads mapped (CPM). One-sided Wilcoxon’s rank-sum test with continuity correction was performed to test the difference of the histidine decarboxylase operon abundance in the patient's sample compared to the healthy control and the p values were labeled with asterisks (ns for p > 0.05; * for p ≤ 0.05; ** for p ≤ 0.01; *** for p ≤ 0.001 and **** for p ≤ 0.0001).
